# NAFLD and Increased Aortic Stiffness: Parallel or Common Physiopathological Mechanisms?

**DOI:** 10.3390/ijms17040460

**Published:** 2016-04-20

**Authors:** Cristiane A. Villela-Nogueira, Nathalie C. Leite, Claudia R. L. Cardoso, Gil F. Salles

**Affiliations:** Department of Internal Medicine, Medical School and University Hospital Clementino Fraga Filho, Universidade Federal do Rio de Janeiro, Rua Croton 72, Rio de Janeiro 22750-240, Brasil; crisvillelanog@gmail.com (C.A.V.-N.); nathaliecleite@gmail.com (N.C.L.); claudiacardoso@hucff.ufrj.br (C.R.L.C.)

**Keywords:** non-alcoholic fatty liver disease, steatohepatitis, liver fibrosis, arterial stiffness, pulse wave velocity

## Abstract

Non-alcoholic fatty liver disease (NAFLD) has become the leading cause of chronic liver diseases worldwide. Liver inflammation and fibrosis related to NAFLD contribute to disease progression and increasing liver-related mortality and morbidity. Increasing data suggest that NAFLD may be linked to atherosclerotic vascular disease independent of other established cardiovascular risk factors. Central arterial stiffness has been recognized as a measure of cumulative cardiovascular risk marker load, and the measure of carotid-femoral pulse wave velocity (cf-PWV) is regarded as the gold standard assessment of aortic stiffness. It has been shown that increased aortic stiffness predicts cardiovascular morbidity and mortality in several clinical settings, including type 2 diabetes mellitus, a well-known condition associated with advanced stages of NAFLD. Furthermore, recently-published studies reported a strong association between NAFLD and increased arterial stiffness, suggesting a possible link in the pathogenesis of atherosclerosis and NAFLD. We sought to review the published data on the associations between NAFLD and aortic stiffness, in order to better understand the interplay between these two conditions and identify possible common physiopathological mechanisms.

## 1. Introduction

Non-alcoholic fatty liver disease (NAFLD) is currently the most prevalent chronic liver disease worldwide and the most frequent cause of abnormal liver enzymes in daily practice [1]. It is clearly related to metabolic syndrome, and its association with progressive liver fibrosis leading to cirrhosis and hepatocellular carcinoma has also been well established [2,3,4,5]. In addition to liver disease, NAFLD is also associated with extrahepatic diseases. Type 2 diabetes mellitus, an increasingly prevalent disease worldwide, is currently regarded as one of NAFLD‘s main risk factors, and it correlates with the severest histological aspects of NAFLD, with a growing prevalence of hepatocellular carcinoma [6,7,8,9]. Furthermore, NAFLD has also been linked to increased cardiovascular risk. A recent meta-analysis showed a 57% increase in overall mortality in patients with NAFLD, not only related to liver disease, but also due to cardiovascular disease (CVD) [10].

Regarding epidemiological aspects, NAFLD affects nearly 20% of the population worldwide, with its highest prevalence being described in South America (35%) and in Middle East (32%) [11]. In patients with associated risk factors, such as morbidly obese patients, NAFLD prevalence can achieve rates as high as 80% [12].

The spectrum of NAFLD encompasses a group of distinct liver diseases. Excluding alcohol ingestion greater than 20 g/day in women and 30 g/day in men and additional specific causes of steatosis, such as drug-induced and malnutrition among others, NAFLD ranges from simple steatosis, defined when at least 5% of hepatocytes are affected by fat; steatohepatitis (NASH), which comprises inflammation with ballooning; and ultimately, fibrosis, evolving to cirrhosis and its complications, such as hepatocellular carcinoma [13,14].

Increased arterial stiffness is an established cardiovascular risk marker in several clinical settings and had been proposed to reflect the cumulative burden of cardiovascular risk factors on the vascular wall [15,16]. Moreover, some recent studies have reported strong associations between increased aortic stiffness and NAFLD, particularly at its more advanced stages [17]. Hence, the aim of this review is to provide a comprehensive overview of previous studies assessing relationships between NAFLD and increased arterial stiffness in order to better understand the interplay between these two conditions and identify possible common physiopathological mechanisms.

## 2. Cardiovascular Risk and Non-Alcoholic Fatty Liver Disease (NAFLD)

Growing evidence has shown that NAFLD may be closely related to atherosclerotic vascular disease over and beyond other well-known cardiovascular risk factors [18,19]. Cardiovascular disease is the most common cause of mortality among patients with NAFLD [20,21]. Kim *et al*., in 4023 individuals without any suspicion of liver disease or coronary artery disease, described that increased coronary artery calcification scores were associated with the presence of NAFLD, independent of traditional risk factors and of visceral adiposity, suggesting that NAFLD might be a risk factor for coronary artery disease [22]. A recent meta-analysis showed that NAFLD was associated with increased carotid-artery intima media thickness, impaired flow-mediated vasodilatation, increased arterial stiffness and increased coronary artery calcification. These associations were all independent of known risk factors and metabolic syndrome traits in a wide range of patient populations [23]. Further, the Framingham Heart study observed, among 3014 participants who performed a multidetector computed tomography (CT)-scan, that there was a significant association between NAFLD and coronary artery calcium and a trend towards a significant association between hepatic steatosis and previous clinical cardiovascular disease [24]. In a cohort of 755 healthy males who performed 18F-fluorodeoxyglucose (FDG) positron emission tomography with computed tomography, patients with NAFLD showed elevated carotid FDG uptake, besides an augmented carotid intima media thickness. These findings hinted that they might be at an increased risk of having inflammatory atherosclerotic plaques in the carotid arteries [25]. Targher *et al.* also demonstrated that patients with steatohepatitis when compared to those with simple steatosis and to controls had a greater carotid artery intima media thickness. Moreover, the same study showed that the histologic severity of nonalcoholic steatohepatitis was also related to carotid artery intima media thickness, regardless of traditional cardiovascular risk factors, insulin resistance and metabolic syndrome elements [26]. In resume, there is well-established evidence of associations between NAFLD and clinical and pre-clinical cardiovascular diseases.

Nevertheless, the physiopathological mechanisms underlying the associations between NAFLD and cardiovascular disease development are much debated, but still largely unsettled. Yoneda *et al.* showed, for the first time, elevated levels of high-sensitivity C-reactive protein (hs-CRP) in patients with biopsy-proven NASH, implying there may be a shared pathway between the severity of liver disease and the levels of hs-CRP, a well-known marker of cardiovascular risk [27]. Some studies observed that intrahepatic messenger RNA expression of C-reactive protein, interleukin-6 or plasminogen activator inhibitor 1 (PAI-1) was associated with the severest forms of NAFLD, mostly steatohepatitis [27,28,29]. Wieckoswska *et al.* correlated interleukin-6 liver expression with plasma levels and liver histology in patients with NASH and diabetes, hinting at a possible link between NAFLD and insulin resistance [28]. Similarly, Thuy *et al.* demonstrated an association between PAI-1, ingestion of a fructose-enriched diet and NAFLD [29]. Cigolini *et al.* also showed that increased PAI-1 was correlated with liver steatosis, implying that it might be mediated by concomitant alterations in plasma triglycerides and insulin concentrations [30]. In the same direction, Targher *et al.* reported that levels of fibrinogen and PAI-1 activity were higher in men with NASH, as well as plasma hs-CRP levels. They also had lower adiponectin levels compared to overweight men without steatosis with comparable visceral adiposity, suggesting that nonalcoholic steatohepatitis may be a factor for a more atherogenic risk profile besides its contribution to visceral adiposity [31]. In this setting, adiponectin concentrations may play a role [32,33,34]. Higher adiponectin levels were associated with a minor risk of myocardial infarction on a nested case control study among 18,225 male participants [33]. Low adiponectin levels are frequently observed in patients with NAFLD. We evaluated cytokine levels in 84 diabetic patients with biopsy-proven NAFLD: patients with NASH or with advanced fibrosis had equal cytokine levels to those without NASH or with absent/light fibrosis, except for lower serum adiponectin levels [34].

## 3. Prognostic Markers: The Role of Fibrosis

The conundrum of NAFLD is to identify patients whose disease will progress and impact survival. The natural history of NAFLD is a dynamic process that has been frequently revised. NAFLD has been considered a stable disease that seldom leads to advanced fibrosis. In a long-term follow-up study, only 1% of patients with simple steatosis presented cirrhosis, whereas among those with NASH, 11% developed cirrhosis and 7.3% died from a liver-related cause after 15.6 years of follow-up [35]. Overall liver-related survival was reduced in Swedish subjects with NAFLD and NASH, particularly in those with significant liver fibrosis, whereas bland steatosis was not associated with any increase in mortality risk, compared to the Swedish general population, followed for a median of 21 years [36]. Thus, current studies support the concept that the presence and severity of liver fibrosis on liver biopsy is the main surrogate marker of long-term prognosis. Hence, it would be important to implement accurate non-invasive markers to identify fibrosis to help to manage high risk patients.

Besides identifying early fibrosis, the recognition of patients who might be at risk for fibrosis progression is of utmost importance in order to define the best management for this specific population. Studies with paired biopsies identified clinical and biochemical aspects that helped in risk stratification regarding fibrosis progression. A recent meta-analysis [37], which included 11 cohort studies with biopsy-proven NAFLD (150 with simple steatosis and 261 with NASH), described that arterial hypertension and a low AST/ALT ratio at baseline predicted liver fibrosis progression. In this meta-analysis, two subgroups of patients were identified according to the rate of fibrosis progression: rapid and slow progressors. The first group comprised 21.1% of patients who had Stage 0 fibrosis at baseline, but in an average of 5.9 years developed fibrosis Stages 3 or 4. The majority of patients were categorized in the second group, which consisted of patients who had low fibrosis progression rate, changing their subsequent biopsies by one or two stages. Two of four studies in the systematic review observed that patients with a higher steatosis grade were more likely to develop progressive fibrosis. Remarkably, in this meta-analysis, no association was found between baseline severity of necroinflammation and risk of progressive fibrosis. This led to the concept that both patients with simple steatosis and with NASH may develop progressive liver fibrosis [37]. However, comparing patients with simple steatosis and NASH at baseline who had no fibrosis at baseline (F0), the rate of fibrosis progression was twice faster in patients with NASH (0.14 *vs.* 0.07 stages). Hence, although fibrosis progression was observed in both groups, it was slower in the simple steatosis group. Nevertheless, these findings differ from those reported in a previous meta-analysis of patients with NASH [38]. It estimated an overall fibrosis progression of 0.03 stages per year, and only age and inflammation on initial biopsy were predictors of progression to advanced fibrosis. Otherwise, in a review of 70 patients with untreated NAFLD who performed two liver biopsies with an interval of more than one year, a significant proportion of patients with NAFLD progressed towards well-defined NASH with bridging fibrosis, especially if metabolic risk factors deteriorated [39]. In this study, even mild inflammation or fibrosis could be considered as prognostic markers, increasing the risk of progression when compared to steatosis alone [39]. It is thus important to define two distinct situations in NAFLD that is simple steatosis, which seems to have a benign course with slower liver fibrosis progression, and steatosis with inflammation that could point to a progressive disease [40].

## 4. Aortic Stiffness and NAFLD

Arterial stiffness is the consequence of a complex interaction between stable and dynamic effects in structural and cellular components of the vascular wall. These vascular changes result from hemodynamic forces and extrinsic factors, like hormones, salt and glucose regulation. Arterial stiffness depends on the structural and geometric properties of the arterial wall and on the distending pressure. Its main determinants are aging and blood pressure [41,42]. Increased arterial stiffness occurs in a heterogeneous pattern predominantly on central segments, sparing peripheral arteries [43]. The stability, resiliency and compliancy properties of the vascular wall rely on two important scaffolding proteins: collagen and elastin. The quantity of such molecules is generally kept stable by a slow, but dynamic interplay of production and degradation. Deregulation of this balance, which may be stimulated by an inflammatory milieu, may lead to overproduction of altered collagen and reduced quantities of normal elastin, leading to increased arterial stiffness [44]. Prevalent diseases, such as arterial hypertension and diabetes mellitus in conjunction with ageing, augment these vascular alterations that worsen artery stiffening in different and synergistic ways. The evaluation of carotid-femoral pulse wave velocity (cf-PWV) can be easily obtained and is regarded as the gold standard method of assessing central aortic stiffness [40]. Further, increased aortic stiffness has been shown to predict cardiovascular morbidity and mortality in individuals with end-stage renal disease [45], hypertension [46], diabetes [47] and in general population-based samples [48,49].

Several previous studies [50,51,52,53,54,55,56,57,58,59,60,61,62,63,64,65], resumed in Table 1, have evaluated the relationships between NAFLD and arterial stiffness. All studies, except two of them [64,65], had cross-sectional designs, and all confirmed an association between increased arterial stiffness and NAFLD (mainly detected by ultrasonography), independent of other traditional cardiometabolic risk factors. Of note, one of these studies [56] demonstrated that the association between NAFLD and increased arterial stiffness was already present at adolescence. In this study, on a 17-year old population cohort from Australia, two groups were categorized according to their metabolic profile as a “high risk” and a “low risk” metabolic cluster. Central PWV was evaluated in both group, and NAFLD was diagnosed by abdominal ultrasound. Males and females with NAFLD in the presence of the metabolic cluster had greater PWV. They concluded that NAFLD was associated with increased arterial stiffness only in the presence of the “high risk” metabolic cluster, suggesting that arterial stiffness associated with NAFLD was linked to the presence of an adverse metabolic profile in adolescents [56]. However, because of their cross-sectional designs, no causal deductions could be drawn, only mere correlations. Of note, only three studies were performed in patients with NAFLD confirmed by histologic evaluation [52,57,62]. Sunbul *et al.* [57] evaluated in 100 biopsy-proven NAFLD patients the relation among arterial stiffness measures and the histological severity of NAFLD and epicardial fat thickness. Among the included patients matched to 50 control individuals, 33% were diabetic, and 55% fulfilled the criteria for metabolic syndrome. Measurements of arterial stiffness using cf-PWV and the augmentation index (AIx) were performed, and epicardial fat thickness was assessed by echocardiography. Patients with NAFLD showed significantly higher aortic PWV (7.0 ± 1.1 *vs.* 6.2 ± 0.8 m/s, *p* < 0.001) and AIx values (22.2% ± 13.1% *vs.* 17.4% ± 12.3%, *p* = 0.02) compared to controls, after adjusting for all potential confounders. Their results corroborated that NAFLD patients had an increased arterial stiffness, which was independently related to the severity of the liver fibrosis and increased epicardial fat thickness [57]. Otherwise, Ozturk *et al.* [62], evaluating 61 biopsy-proven NAFLD patients and 40 matched controls, found significant associations between NAFLD and aortic stiffness, independent of the presence of metabolic syndrome; but no correlation with histological liver fibrosis or inflammatory activity. Chen *et al.* [60] also described the association of advanced fibrosis with subclinical atherosclerosis in 2550 participants with ultrasound-diagnosed NAFLD. In this study, the NAFLD fibrosis score was calculated to assess the severity of the fibrosis of NAFLD patients. An NAFLD score >0.676 indicated the presence of advanced fibrosis in their study. The indicators of early atherosclerosis in the study were the carotid intima media thickness, carotid plaques and brachial-ankle pulse wave velocity (ba-PWV). They found that advanced fibrosis indicated by the NAFLD score was associated with carotid intima media thickness, with the presence of carotid plaques and with increased arterial stiffness, independent of usual cardiometabolic risk factors and insulin resistance [60]. There are only two longitudinal studies [64,65] evaluating the progression of arterial stiffness and the presence of NAFLD. The first one [64], with two arterial stiffness evaluations, employed brachial-ankle PWV, hence measuring principally peripheral arterial stiffness. It was accomplished in 1225 individuals on a five-year follow-up. This study concluded that individuals with NAFLD at first evaluation (diagnosed by ultrasonography) had a faster arterial stiffening than individuals without NAFLD, regardless of the concomitance of metabolic syndrome. We [65] performed serial cf-PWV measurements and evaluated liver fibrosis by transient elastography in 291 diabetic patients with NAFLD over a median follow-up of seven years. We observed that both a high aortic stiffness at the second cf-PWV examination (odds ratio (OR): 3.0; 95% confidence interval (CI): 1.3–7.2; *p* = 0.011) and a further augment in aortic stiffness (OR: 2.1; 95% CI: 1.0–4.3; *p* = 0.046) pointed to the increased likelihood of presenting advanced liver fibrosis on transient elastography examination [65]. Thus, it is possible that the chronological longitudinal associations between NAFLD and arterial stiffness may be bidirectional: NAFLD may hasten arterial stiffness progression, whilst increasing aortic stiffness may lead prior NAFLD in the direction of advanced liver fibrosis [65].

## 5. NAFLD and Arterial Stiffness: Is There an Interplay?

Many studies evaluated if NAFLD contributed to other outcomes, such as cardiovascular mortality; and most of them demonstrated an association, but no causality could be shown [20]. Liver disease and atherogenesis might be mediated by inflamed visceral adipose tissue. In this scenario, the liver might play a role of both the target of the resulting systemic abnormalities and as the source of many proatherogenic variables. In this setting, nonalcoholic steatohepatitis might contribute to the pathogenesis of cardiovascular disease in two ways: first, through the systemic release of several inflammatory, prothrombotic and oxidative-stress substances and, second, through the contribution of nonalcoholic fatty liver disease to insulin resistance and atherogenic dyslipidemia.

Insulin resistance is the utmost important factor that triggers the development of NAFLD. This notwithstanding, insulin resistance is probably one of the mechanisms that is also linked to increased arterial stiffness. Both chronic hyperglycemia, as well as hyperinsulinemia have been demonstrated to increase the local activity of the renin-angiotensin-aldosterone system and also the expression of the angiotensin type I receptor in the vascular milieu, leading to hypertrophy of vascular wall and fibrosis [66,67,68]. Due to insulin resistance, the proliferative effects of hyperinsulinemia prevails and promotes an impairment of phosphatidylinositol 3 (PI3)-kinase-dependent signaling responsible for the acute metabolic effects of insulin; still preserving the activity of growth promoting mitogen-activated kinase pathways [69]. Triglyceride in the liver has been considered as an epiphenomenon being a marker of a dysmetabolic state, not adding directly to the genesis of the extrahepatic manifestations of this complication.

Omelchenko *et al.* evaluated the relation between the levels of adiponectin and arterial stiffness parameters using pulse wave velocity (PWV) and the arterial augmentation index (Aix) in NAFLD patients [58]. In their study, adiponectin was positively correlated with Aix (*r* = 0.467; *p* < 0.0001) and with PWV (*r* = 0.348; *p* = 0.011), in spite of a weak correlation coefficient. In a multiple linear regression analysis, adiponectin persisted as a significant predictor of abnormal PWV after controlling for age and gender, suggesting an active role of adiponectin in the pathophysiology of vascular disease in NAFLD patients [58]. Remarkably, it was observed by Kim *et al.* [53] that NAFLD and arterial stiffness have been related even in the absence of arterial hypertension, diabetes and metabolic syndrome. Abdominal ultrasound and brachial-ankle pulse wave velocity (ba-PWV) were investigated in 4467 individuals. NAFLD individuals were classified in non-NAFLD, mild and moderate-to-severe NAFLD groups, respectively. The NAFLD group had higher levels of ba-PWV. NAFLD was independently associated with increased ba-PWV (≥1366 cm/s), independent of multiple covariates (OR: 1.24 and 95% CI: 1.05–1.46). Subgroup analyses revealed that there was a significant association between NAFLD and increased ba-PWV only in individuals without metabolic syndrome (OR: 1.27 and 95% CI: 1.07–1.51). The multivariate linear regression models for the overall study population and for individuals without metabolic syndrome also showed a significant association between NAFLD and the absolute values of ba-PWV; however, the result for individuals with metabolic syndrome did not demonstrate an association [53]. This might point to the possibility that NAFLD pathogenetic mechanism per se could be linked to abnormal arterial stiffness not requiring the coexistence of metabolic syndrome for its occurrence. Recently, Chou *et al.* [61] investigated 4860 subjects who were categorized into normal glucose tolerance, pre-diabetes and newly-diagnosed diabetes groups and, after excluding known diabetes, the independent relationship between non-alcoholic fatty liver disease and arterial stiffness. The severity of non-alcoholic fatty liver disease was divided into mild and moderate-to-severe. Increased arterial stiffness was defined as brachial-ankle pulse wave velocity (ba-PWV) >1400 cm/s. They concluded that the effect of NAFLD on arterial stiffness was apparent in subjects with normal glucose tolerance, but not in diabetes and pre-diabetes [61].

In resume, the possible biological mechanisms linking NAFLD and increased arterial stiffness remain largely unknown, but possibly involve common pathways of chronic low-grade inflammation and adipokines imbalance [70,71]. More prospective studies, including diabetic and non-diabetic patients, are necessary to investigate whether there are causal relationships between them. On the other hand, aortic stiffness, ideally measured by carotid-femoral PWV, may be a useful tool to identify high-risk patients concerning both cardiovascular and liver disease. Its use as a prognostic marker may help define better strategies to slow the progression of both liver and cardiovascular disease. In the future, prospective studies with serial PWV and liver disease severity evaluation may confirm its utility in assessing improvement in both scenarios′ outcomes.

## Figures and Tables

**Table 1:** Studies evaluating associations between non-alcoholic fatty liver disease (NAFLD) and arterial stiffness.

Author, Year	Number of Participants and Methods of Liver Investigation	Study Design	Aims	Conclusions
Shiotani *et al.*, 2005 [50]	353 young university Japanese adults, submitted to abdominal ultrasound.	Transversal	To evaluate the validity of noninvasive ba-PWV measurements in overweight young adults.	ba-PWV was increased in males with NAFLD and might conceivably be useful to predict NAFLD.
Salvi *et al.*, 2010 [51]	220 participants (123 women), aged between 30 and 70 years, from the Cardio-gambettola observatory liver steatosis estimation (GOOSE) study, submitted to abdominal ultrasound.	Transversal	To evaluate the relationship between metabolic syndrome, NAFLD and subclinical vascular disease, evaluated by carotid IMT and cf-PWV.	A possible independent role of NAFLD in determining arterial stiffness.
Vlachopoulos *et al.*, 2010 [52]	23 biopsy-proven NAFLD patients and 28 matched controls.	Transversal	To investigate associations between NAFLD and functional arterial changes and early atherosclerosis.	NAFLD was associated with endothelial dysfunction and aortic stiffness (cf-PWV).
Kim *et al*., 2012 [53]	4467 patients submitted to abdominal ultrasound.	Transversal	To evaluate the association of NAFLD and ba-PWV in patients with and without metabolic syndrome.	NAFLD was independently associated with increased ba-PWV, irrespective of multiple covariates, only in patients without metabolic syndrome.
Huang *et al.*, 2012 [54]	8632 Chinese from a population-based sample; NAFLD detected by ultrasound.	Transversal	To evaluate associations between NAFLD and early atherosclerosis (carotid IMT and ba-PWV).	NAFLD was associated with increased carotid IMT and ba-PWV, independent of traditional CV risk factors and metabolic syndrome.
Lee *et al.*, 2012 [55]	1442 healthy adults; NAFLD detected by ultrasound.	Transversal	To evaluate association between NAFLD and arterial stiffness (ba-PWV).	Arterial stiffness was associated with NAFD, independent of classical CV risk factors.
Huang *et al.*, 2013 [56]	964 adolescents (17-year-olds) from an Australian birth cohort, submitted to abdominal ultrasound.	Transversal	To examine if NAFLD was associated with aortic PWV, independent of cardiometabolic factors.	Aortic PWV was related to the presence of NAFLD that was predicated by the presence of an adverse metabolic profile in adolescents.
Sunbul *et al.*, 2014 [57]	100 patients with biopsy-proven NAFLD and 50 age- and sex-matched controls.	Transversal	To examine the relationship between aortic PWV and AIx, the histological severity of NAFLD and epicardial fat thickness (EFT).	Patients with NAFLD have an increased arterial stiffness, which reflects both the severity of liver fibrosis and increased EFT values.
Omelchenko *et al.*, 2014 [58]	52 NAFLD patients detected by ultrasound.	Transversal	To evaluate associations between adiponectin levels and arterial stiffness parameters (cf-PWV and AIx).	Adiponectin remained a significant predictor of PWV, even after controlling for age and gender, suggesting an active role of adiponectin in the pathophysiology of vascular disease in NAFLD patients.
Yu *et al.*, 2014 [59]	1296 non-obese, non-hypertensive, non-diabetic adults, NAFLD by ultrasound.	Transversal	To evaluate then association between NAFLD and arterial stiffness (ba-PWV).	NAFLD was associated with ba-PWV in Chinese individuals without obesity, hypertension and diabetes.
Chen *et al.*, 2015 [60]	2550 participants with ultrasound-confirmed NAFLD from a community-based sample.	Transversal	To evaluate whether advanced fibrosis assessed by NAFLD fibrosis score was associated with subclinical atherosclerosis in NAFLD patients.	Advanced fibrosis was associated with carotid intima media thickness, the presence of carotid plaques and arterial stiffness, independent of cardiometabolic risk factors and insulin resistance.
Chou *et al.*, 2015 [61]	4860 non-diabetic, pre-diabetic and newly-diagnosed T2DM individuals, evaluated by abdominal ultrasound.	Transversal	To evaluate PWV in patients with NAFLD.	The effect of NAFLD on arterial stiffness was apparent only in subjects with normal glucose tolerance.
Ozturk *et al.*, 2015 [62]	61 biopsy-proven NAFLD patients and 41 controls without NAFLD; adult male patients between 20 and 40 years of age.	Transversal	To evaluate the relationship between NAFLD and subclinical atherosclerosis and to investigate the associations according to the presence or absence of metabolic syndrome.	The presence of NAFLD was associated with endothelial dysfunction and atherosclerosis, independent of metabolic syndrome.
Chung *et al.*, 2015 [63]	2954 healthy individuals; NAFLD detected by ultrasound.	Transversal	To evaluate the association between NAFLD and arterial stiffness (cardio-ankle vascular index).	NAFLD was associated with increased arterial stiffness, independent of cardio-metabolic risk factors.
Li *et al.*, 2015 [64]	728 men and 497 women without hypertension and diabetes; NAFLD detected by ultrasound.	Longitudinal	To evaluate the relationship between the presence of NAFLD at baseline and progression of arterial stiffness (ba-PWV) during follow-up (5 years).	Patients with NAFLD had a faster progression of arterial stiffness, independent of other CV risk factors.
Leite *et al.*, 2015 [65]	291 T2DM patients; NAFLD by abdominal ultrasound or liver biopsy.	Longitudinal	To evaluate the association between progressions of aortic PWV (7 years of follow-up) with advanced liver fibrosis identified by transient elastography.	High or increasing aortic stiffness predicted the development of advanced liver fibrosis on transient elastography.

Abbreviations: T2DM, type-2 diabetes mellitus; NAFLD, non-alcoholic fatty liver disease; cf-PWV, carotid-femoral pulse-wave velocity; ba-PWV, brachial-ankle pulse wave velocity; AIx, arterial augmentation index; IMT, intima media thickness.
